# Synthesis of 2-phenyl-5,6,7,8-tetrahydroquinoxaline derivatives and screening for P2X1-purinoceptor antagonist activity in isolated preparations of rat vas deferens, for translation into a male contraceptive^†^

**DOI:** 10.1093/biolre/ioaa117

**Published:** 2020-07-10

**Authors:** Mitch Mathiew, Belinda M Dennis, Felix Bennetts, N N Eunice Su, Nghi Nguyen, Antony Botteon, Jonathan B Baell, Sabatino Ventura

**Affiliations:** 1 Medicinal Chemistry, Monash Institute of Pharmaceutical Sciences, Monash University, Parkville, Victoria. Australia; 2 Drug Discovery Biology, Monash Institute of Pharmaceutical Sciences, Monash University, Parkville, Victoria. Australia

**Keywords:** adenosine-5′-triphosphate (ATP), male reproductive tract, noradrenaline, sperm transport, sympathetic neurotransmission

## Abstract

Sympathetically mediated contractions of smooth muscle cells in the vasa deferentia are mediated by neuronally released adenosine 5′-triphosphate (ATP) and noradrenaline, which stimulate P2X1-purinoceptors and α_1A_-adrenoceptors, respectively. This process is crucial for sperm transport, as demonstrated in knockout mouse studies where simultaneous genetic deletion of P2X1-purinoceptors and α_1A_-adrenoceptors resulted in male infertility. We hypothesize that dual pharmacological antagonism of these two receptors could inhibit sperm transport sufficiently to provide a novel nonhormonal method of male contraception. To generate a suitable P2X1-purinoceptor antagonist, substituents were introduced on the phenyl moiety of 2-phenyl-5,6,7,8-tetrahydroquinoxaline to create a series of analogues that were tested for P2X1-purinoceptor antagonism in isolated preparations of rat vas deferens. Novel compounds were initially screened for their ability to attenuate contractile responses to electrical field stimulation (EFS: 60 V, 0.5 ms, 0.2 Hz). The addition of polar substituents to the meta, but not ortho, position markedly increased the inhibition of contractions, as did the addition of both polar and aliphatic substituents to the para position. Di-substituted compounds were also synthesized and tested, resulting in a compound 31 (2-hydroxy, 4-fluoro), which exhibited the greatest potency, with an IC_50_ of 14 μM (95% confidence limits: 12–16 μM). Additionally, compound 31 noncompetitively antagonized contractions mediated by exogenously administered αß-methylene ATP (10 nM–30 μM) but had no inhibitory effect on contractions mediated by exogenously administered noradrenaline (30 nM–100 μM) or acetylcholine (30 nM–100 μM). These results have contributed to a structure–activity relationship profile for the P2X1-purinoceptor that will inform future designs of more potent antagonists.

## Teaser

Novel small molecule P2X1-purinoceptor antagonists were able to selectively inhibit the purinergic component of contractions of the rat vas deferens.

## Graphical Abstract

**Graphical Abstract:**
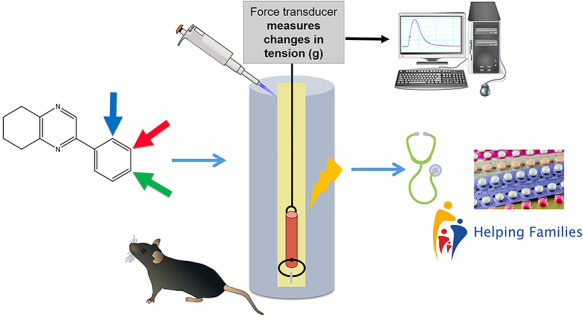

## Introduction

Male contraceptive options are currently limited only to condoms and vasectomies [1]. Condoms prevent sperm entry to the vagina/cervix, thus inhibiting fertilization. Nevertheless, they have a high typical-use annual failure rate of ~15%. Other disadvantages include disruption of foreplay and reduced sensitivity during intercourse [2]. Vasectomies inhibit sperm transport via a surgical procedure in which the vasa deferentia are severed and sealed. Vasectomies are incredibly effective; however, they are not readily reversible and, hence, are not acceptable for men who may wish to have children in the future [3–6]. Based on these limitations, it is clear that there is an unmet need for better, safe, effective, and easy-to-use contraceptives for males.

The acceptability of male contraceptive methods has been widely debated. Several recent surveys have addressed this issue, finding a general willingness of males to take more responsibility regarding contraceptive use and a preparedness of women to trust male partners to do so [3,7–9].

Currently, several hormonal male contraceptives are in development, but will likely have intolerable sexual, behavioral, physiological, and psychological side effects [6]. In addition, several specific male proteins involved in spermatogenesis have been suggested as targets for male contraception [5,10–14]. However, inhibiting these proteins is difficult to reverse, and the contraceptive agents would need to cross the blood–testis barrier to access their site of action [3]. These and most other nonhormonal studies have concentrated on rendering sperm dysfunctional [15–19]. Such contraceptive targets face further biological challenges, including the difficulty of suppressing/altering the function of the large number of spermatozoa produced by men (~1000 per second), compared to one ovum per month in females, and the concern that genetics of offspring could be altered by affecting the production of germline cells. New contraceptives also face the usual issues in the development of pharmaceuticals, including regulatory hurdles, large financial expenditure, preference for oral availability, and unexpected adverse effects [3,20].

The vasa deferentia serve as the conduits between the cauda epididymides and the ejaculatory duct. Powerful, sympathetically mediated contractions of smooth muscle cells surrounding the vasa deferentia propel sperm from their storage site in the cauda epididymis, to the urethra, where it is expelled along with seminal and prostatic fluid [21]. These contractions are mediated by the activation of P2X1-purinoceptor ligand-gated ion channels and α_1A_-adrenergic G protein-coupled receptors (adrenoceptors) by adenosine-5′-triphosphate (ATP) and noradrenaline, respectively [22–24]. Given the integral role of the vas deferens in sperm expulsion, and the effectiveness of vasectomies, pharmacological inhibition of vas deferens contractility has been suggested as a novel male contraceptive target [20,25].

Dual genetic deletion of P2X1-purinoceptors and α_1A_-adrenoceptors in male mice was previously demonstrated to result in 100% infertility while having no adverse effects on behavior or physiology [20]. Additionally, sperm function and quality were seemingly unaffected, as intracytoplasmic injection of sperm taken from the cauda epididymides of these double knockout male mice into wildtype mice ova resulted in fertilization, suggesting easy reversibility. Furthermore, fertilized embryos were implanted into foster mothers, and normal, viable offspring were produced [20].

We hypothesize that dual pharmacological antagonism of P2X1-purinoceptors and α_1A_-adrenoceptors could inhibit sperm transport and provide a readily reversible, orally available male contraceptive, overcoming the various issues associated with hormonal or spermatogenic targets.

**Figure 1:**
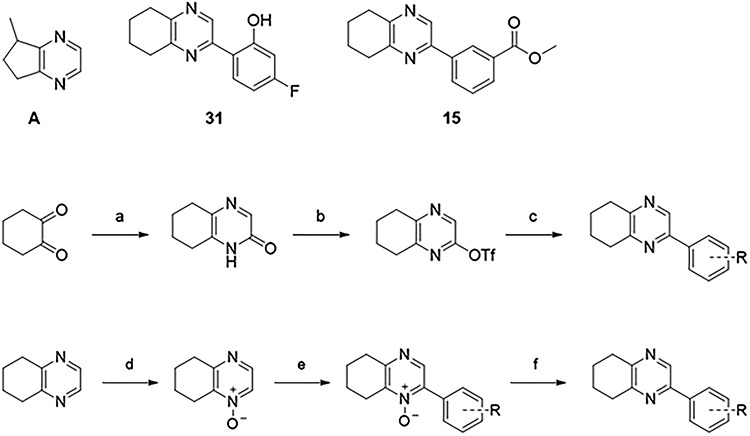
Chemical structures of compound A, compound 31, and compound 15. Preparation of 2-aryl-5,6,7,8-tetrahydroquinoxalines. Reagents and conditions: (a) Glycinamide, aq NaOH, MeOH -30 °C then −10 °C; (b) PhNTf_2_, DMAP, NEt_3_, CH_2_Cl_2_, rt; (c) Pd(PPh_3_)_4_, Cs_2_CO_3_, ArB(OH)_2_ or ArBPin, dioxane, water, 80 °C; (d) mCPBA, CH_2_Cl_2_, 0 °C then rt; (e) Pd(OAc)_2_, HP(*t*-Bu)_3_BF_4_, K_2_CO_3_, ArBr, dioxane, 110 °C; (f) NH_4_CO_2_H, Pd/C, MeOH, rt.

Due to the widespread and long-term safe use of α_1A_-adrenoceptor antagonists, such as tamsulosin, in the treatment of chronic diseases including benign prostatic hyperplasia [26], our studies focus primarily on the development of a suitable P2X1-purinoceptor antagonist, of which no potent and selective options currently exist for use in vivo [25]. Pilot studies in our laboratory have previously shown 2-phenyl-5,6,7,8-tetrahydroquinoxaline to be an antagonist at P2X1-purinoceptors with an IC_50_ of ~134 (56 -) μM (Compound 1: Table 2). Compound 1 was identified from screening a focused set of structurally similar compounds based on 5-methyl-6,7-dihydro-5*H*-cyclopentapyrazine, which was reported in a patent with data suggesting that it was a P2X-purinoceptor antagonist (Compound A: Figure 1) [27]. By altering substituents on the phenyl moiety, a series of analogues of 2-phenyl-5,6,7,8-tetrahydroquinoxaline were synthesized and tested for P2X1-purinoceptor activity in isolated rat vasa deferentia with the aim of increasing potency to a level that was suitable for preclinical testing.

## Materials and methods

### Chemical synthesis protocols

Cyclohexadione was cyclocondensed with glycinamide under basic conditions to form 5,6,7,8-tetrahydroquinoxalin-2(1*H*)-one. Triflation of the bicyclic ketone afforded the key intermediate that furnished the arylated tetrahydroquinoxalines under palladium-catalyzed Suzuki Coupling conditions (Figure 1).

Alternatively, arylation of 5,6,7,8-tetrahydroquinoxaline *N*-oxide could be performed under palladium catalysis, following which, reduction of the *N*-oxide moiety afforded the final structures (Figure 1).

Detailed chemical methods are described in supplementary material.

### Animal ethics and housing

All procedures involving animals were approved by the Monash University Standing Committee for Animal Ethics in Animal Experimentation (ethics number: MIPS2018–14149) and conformed to the code of practice for the care and use of animals for scientific purposes according to the National Health and Medical Research Council, Australia. Animals were purchased from the Monash Animal Research Platform (MARP). Prior to experimentation animals were housed at the Monash Parkville Animal House where they were maintained at 22°C and had access to food and water ad libitum. Their holding room was kept on a 12-h light–dark cycle.

### Isolated rat vas deferens preparation

Adult male Sprague Dawley rats (7–8 weeks old) were euthanized by CO_2_ asphyxiation. The urogenital tract was exposed via a lower abdominal incision, and the vasa deferentia were carefully dissected out and placed into specimen jars containing Krebs–Henseleit solution (NaCl, 118 mM; KCl, 4.69 mM, MgSO_4_.7H_2_O, 1.10 mM; KH_2_PO_4_, 1.18 mM; NaHCO_3_, 25.0 mM; glucose, 11.7 mM; CaCl_2_, 2.50 mM; carbogenated to pH 7.4). Longitudinal segments (1.5–2.0 cm) were taken from each vas deferens, and excess adipose tissue was removed. Segments from the urethral end were used in experiments focussing on P2X1-purinoceptors, and segments from the epididymal end were used in experiments focussing on α_1A_-adrenoceptors, due to relative receptor expression in these regions [28]. For experiments focussing on muscarinic receptors, the mid portion of vas deferens was used.

### Isolated organ bath setup

Vasa deferentia segments were mounted vertically on perspex tissue holders so that contractions of the longitudinal muscle could be measured. The tissue holders were placed in separate 10-mL water-jacketed standard organ baths filled with Krebs–Henseleit solution maintained at 37°C and supplied with carbogen (95% oxygen; 5% CO_2_) gas. Each tissue holder incorporated two platinum electrodes, which were connected to a Grass S88 stimulator (Grass Instruments, MA, USA). The epididymal end of the tissue was tied off and fastened to the tissue holders, and a needle was used to thread cotton ligature through the lumen and tissue wall at the prostatic end of the tissue, which was then attached to a FT03 force displacement transducer (Grass instruments, MA, USA), connected to a ML118 QUAD Bridge (ADInstruments, Australia). The signal was digitized and sent to a personal computer using a ML750 PowerLab/4SP (ADInstruments, Castle Hill, Australia), and data was recorded using LabChart software (version: Chart5 for Windows). The initial tension of the tissues was set at 1.0 g, and tissues were allowed to equilibrate for 1 h under electrical field stimulation (EFS, parameters: pulse duration = 0.5 ms; voltage = 60 V; frequency = 0.01 Hz) prior to experimentation.

### Inhibitory cumulative concentration–response curves

Following the 1-h equilibration period, tissues were allowed to rest for 10 min before washing with Krebs–Henseleit solution and the readjustment of tension to 1.0 g. Cumulative concentration–response curves were constructed for each test compound to assess their inhibitory effects on contractile responses of the isolated rat vas deferens to EFS (parameters: pulse duration = 0.5 ms; voltage = 60 V; frequency = 0.2 Hz). Contractions elicited by these stimulation parameters were abolished by the noradrenergic neuron blocking drug, guanethidine (10 μM). About 10 min was allowed for the magnitude of the control contractile responses to stabilize before cumulative addition to the organ baths of increasing concentrations of test compounds (1–300 μM) or the corresponding vehicle control. A concentration progression ratio of half a log unit was used. Subsequent concentrations were added when the response to the previous concentration had plateaued (5–10 min) or after 10 min if no effect was observed. After the contractile responses in the presence of the final concentration had plateaued, tissues were washed thoroughly with Krebs–Henseleit solution (three washes with approximately 10 times the bath volume each, with approximately 10 min between washes) to test for reversibility of test compound-induced inhibition of contractility. In selected experiments the washing step was replaced with the addition of 300 nM prazosin (an α_1A_-adrenoceptor antagonist), to confirm that the test compound was inhibiting the purinergic and not the noradrenergic component of EFS-induced contractions.

### Agonist discrete concentration–response curves

In a separate set of experiments, test compounds with a half maximal inhibitory concentration (IC_50_) ≤ 25 μM underwent further testing, to investigate receptor specificity and assess the nature of antagonism. Isolated preparations of rat vas deferens were set up as previously described. However, following the 1-h equilibration period, tissues were allowed to rest for 15 min before washing with Krebs–Henseleit solution and the readjustment of tension to 1.0 g. Discrete agonist concentration–response curves were then constructed to exogenously administered αß-methylene ATP, noradrenaline, or acetylcholine (P2X1-purinoceptor, α_1A_-adrenoceptor, and muscarinic receptor agonists, respectively), on unstimulated tissues using a concentration progression ratio of half a log unit. The tissues were washed thoroughly with Krebs solution (for at least 30 s) once the contractile responses had peaked following the addition of each agonist concentration (or after 10 s if no contractile response was seen). Agonist additions were applied at least 15 min apart to avoid receptor desensitization. After the addition and washout of the final agonist concentration, 30 μM of the test compound (or corresponding vehicle control) was added to each organ bath and allowed to incubate for 1 h. Following this incubation, tissues were washed with Krebs–Henseleit solution and their tensions readjusted to 1.0 g if required, and the test compound/vehicle control was re-added. A second discrete concentration–response curve was then constructed, with the test compound/vehicle control being re-added immediately after the washing out of each agonist concentration.

For assays in which noradrenaline or acetylcholine was the exogenously added agonist, a priming dose of 10 μM of agonist was added following the 15-min rest after the initial 1-h equilibration period. This priming dose was washed out after the tissue reached maximum contraction (or after 10 s), and the tissue was allowed to rest for a further 30 min before the tension was adjusted to 1.0 g and the construction of the first agonist concentration–response curve commenced.

### Data handling, statistical analysis, and go/no go criteria

All data points shown on the graphs are mean ± standard error of the mean (SEM) of at least four separate experiments where *n* = the number of animals used. GraphPad Prism Software (version 7.0) (GraphPad Software, USA) was used to produce all graphs and perform all statistical tests. *P*$\le$ 0.05 was used to define statistical significance.

Initial screens of novel compounds for their ability to inhibit EFS-induced contractions of isolated rat vas deferens were analyzed by two-way repeated measures analysis of variance (ANOVA). This test was used to determine whether the inhibitory cumulative concentration–response curves in the presence of test compounds were different to their corresponding vehicle controls. The *P* value calculated for the interaction between treatment and concentration was used to determine significance. If and when inhibition of EFS-induced contractions by test compounds was greater than 50% of the initial control contraction and greater than 15% of the inhibition produced by the corresponding vehicle control, the potency of test compounds was expressed in terms of IC_50_ values (the concentration required to inhibit initial control contractions by 50%). IC_50_ values were determined by fitting nonlinear regression curves using a log (inhibitor) vs. response (three parameters) slope with a confidence interval of 95% (the confidence limits are expressed in parentheses with the corresponding IC_50_ values in the Results section). In some cases the upper or both 95% confidence limits are not given due to the GraphPad software being unable to calculate a value.

Concentration–response curves to αβ-methylene ATP were only conducted on test compounds that yielded an IC_50_ of <100 μM to confirm activity at P2X1-purinoceptors and investigate the nature of antagonism. Two-way repeated measures ANOVA was also used to analyze agonist concentration–response curves to see whether contractile responses before and after incubation with test compounds (or a corresponding vehicle control) were different. If and when concentration–response curves to αβ-methylene ATP were shifted to the right in a parallel fashion suggesting competitive antagonism, a pK_B_ estimate for the test compound was calculated. pK_B_ estimates were calculated by determining a concentration ratio by dividing the EC_50_ for αβ-methylene ATP in the presence of the test compound by the EC_50_ for αβ-methylene ATP in the absence of the test compound. K_B_ was then estimated by dividing the concentration of the test compound used (30 μM) by the concentration ratio − 1.

Two-tailed Student *t*-tests were used to analyze the effect of the addition of prazosin to organ baths on EFS-induced contractions containing either a test compound (300 μM) or a corresponding vehicle control.

### Synthetic chemistry, dilutions, and reagents

Thirty-three new test compounds were synthesized. Derivatives of 2-phenyl-5,6,7,8-tetrahydroquinoxaline with different functional groups on the phenyl ring were synthesized. See Table 1 for detailed information on all test compounds which were synthesized and tested.

**Table 1:** Structure of 2-phenyl-5,6,7,8-tetrahydroquinoxaline shown with R groups in the ortho, meta, and para positions and in combination (from left to right).

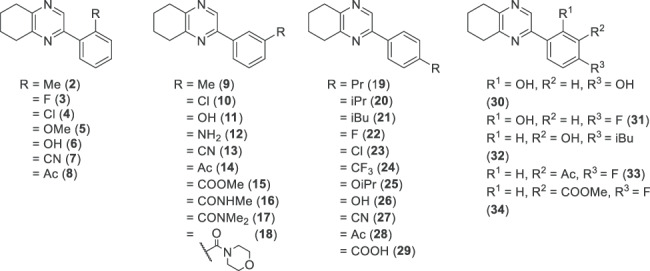

100-mM stock solutions of the test compounds were made up in 100% dimethyl sulfoxide (DMSO). Serial dilutions were performed on the day of experimentation using MilliQ water and DMSO as required to reach the desired concentration. The volumes of MilliQ water and DMSO used were dependent on the solubility of each individual test compound, and vehicle solutions were made up containing the same ratio of the two solvents as used to dissolve and dilute each test compound.

αß-Methylene ATP, prazosin, and acetylcholine were dissolved and diluted in MilliQ water, while noradrenaline was dissolved and diluted in a catecholamine diluent (NaCl, 154 mM; NaH_2_PO_4_·H_2_O, 1.2 mM; ascorbic acid, 0.2 mM).

Acetylcholine chloride, dimethyl sulfoxide (DMSO), αß-methyleneadenosine 5′-triphosphate lithium salt, norepinephrine bitartrate salt, and prazosin hydrochloride were purchased from Sigma-Aldrich.

## Results

### Inhibitory cumulative concentration–response curves

All 33 new compounds tested decreased EFS-induced contractions in isolated rat vas deferens preparations in a concentration-dependent manner. IC_50_ values of test compounds for inhibition of EFS-induced contractility ranged from 14 to 214 μM (see Table 2).

**Table 2:** SAR of 2-aryl-5,6,7,8-tetrahydroquinoxaline against bioassay of EFS (60 V, 0.5 ms, 0.2 Hz)-induced contractility of isolated rat vas deferens preparations.

**Compound number**	R (Ortho)	R (Meta)	R (Para)	EFS Max % decrease	IC_50_ (μM)
**1**	H	H	H	18	133 (56-)
**2**	Me	H	H	57	214 (156–294)
**3**	F	H	H	-	-
**4**	Cl	H	H	32	115 (−)
**5**	OMe	H	H	-	-
**6**	OH	H	H	25	54 (45–64)
**7**	CN	H	H	-	-
**8**	Ac	H	H	-	-
**9**	H	Me	H	-	-
**10**	H	Cl	H	-	-
**11**	H	OH	H	36	24 (19–30)
**12**	H	NH_2_	H	28	221 (165-)
**13**	H	CN	H	19	210 (158-)
**14**	H	Ac	H	28	74 (56–97)
**15**	H	CO_2_Me	H	22	23 (18–29)
**16**	H	CONHMe	H	28	87 (67–115)
**17**	H	CONMe_2_	H	28	78 (6–112)
**18**	H	FX3	H	-	-
**19**	H	H	Pr	-	-
**20**	H	H	*i*-Pr	53	113 (70–183)
**21**	H	H	*i*-Bu	52	47 (31–70)
**22**	H	H	F	28	44 (30–64)
**23**	H	H	Cl	-	-
**24**	H	H	CF_3_	-	-
**25**	H	H	O*i*-Pr	48	204 (94–445)
**26**	H	H	OH	18	31 (22–43)
**27**	H	H	CN	13	43 (30–58)
**28**	H	H	Ac	-	-
**29**	H	H	CO2H	15	27 (18–40)
**30**	OH	H	OH	21	43 (31–60)
**31**	OH	H	F	13	14 (12–16)
**32**	H	OH	*i*-Bu	8	24 (20–29)
**33**	H	Ac	F	-	-
**34**	H	CO_2_Me	F	-	-

Lead optimization resulted in the synthesis of a focused library of analogues exploring the structure–activity relationship (SAR) of this series. The effects of a variety of substituents at the ortho, meta, and para positions of the RHS aryl ring were explored based on the 2-phenyl-5,6,7,8-quinoxaline previously discovered in our laboratory (IC_50_ = 133 (56 -) μM). These substituents include various halogens and electron-withdrawing and electron-donating groups. Groups that were identified as having increased potency were then examined to see if there were cooperative SAR effects.

### Effect of ortho-substituted test compounds on EFS-induced contractions

Initial optimization efforts focused on introducing substituents to compound **1** in the ortho position (Table 1). Derivatives with a fluoro (**3**), methoxy (**5**), cyano (**7**), or acetyl (**8**) substituent did not result in a decrease in contractility compared to the corresponding vehicle control (Table 2). In contrast, derivatives with a chloro (**4**, IC_50_ = 115 (−) μM) or methyl (**2**, IC_50_ = 214 (156–294) μM) had similar activity as compound **1** in decreasing EFS-induced vas deferens contractility. Introduction of a hydroxy (**6**, IC_50_ = 54 (45–64) μM) substituent, however, resulted in an almost 3-fold increase in activity (Table 2).

### Effect of meta-substituted test compounds on EFS-induced contractions

Exploration of substituents in the meta position was subsequently carried out. Incorporation of methyl (**9**), chloro (**10**), or morpholinocarbonyl (**18**) substituent led to complete loss of activity (Table 2). Introduction of a cyano (**13**, IC_50_ = 210 (158 -) μM) or amino (**12**, IC_50_ = 221 (165 -) μM) functional group caused a slight loss in activity relative to compound **1**. In contrast, introduction of a hydroxy (**11**, IC_50_ = 24 (19–30) μM) functional group resulted in improved inhibitory activity, leading to a 5-fold increase in potency. Similarly, introduction of a carbonyl substitution such as acetyl (**14**, IC_50_ = 74 (56–97) μM), methoxycarbonyl (**15**, IC_50_ = 23 (18–29) μM, Figure 1), methylbenzamide (**16**, IC_50_ = 87 (67–115) μM), or dimethyl benzamide (**17**, IC_50_ = 78 (6–112) μM) resulted in up to a 5-fold increase in activity (Table 2).

### Effect of para-substituted test compounds on EFS-induced contractions

Examination of substituents in the para position revealed that the propyl (**19**), chloro (**23**), trifluoromethyl (**24**), and acetyl (**28**) derivatives were inactive. In contrast, fluoro (**22**, IC_50_ = 44 (30–64) μM) and cyano (**27**, IC_50_ = 43 (30–58) μM) both gave a 3-fold improvement in activity. Similarly, other polar groups such as hydroxy (**26**, IC_50_ = 31 (22–43) μM) and carboxy (**29**, IC_50_ = 27 (18–40) μM) provided a 4-fold improvement in activity. Introduction of branched alkyl groups such as an isopropyl (**20**, IC_50_ = 113 (70–183) μM) or isobutyl (**21**, IC_50_ = 47 (31–70) μM) also resulted in an increase in activity, with isobutyl being the most potent, providing a 3-fold improvement in the IC_50_ (Table 2).

### Effect of di-substituted test compounds on EFS-induced contractions

**Figure 2:**
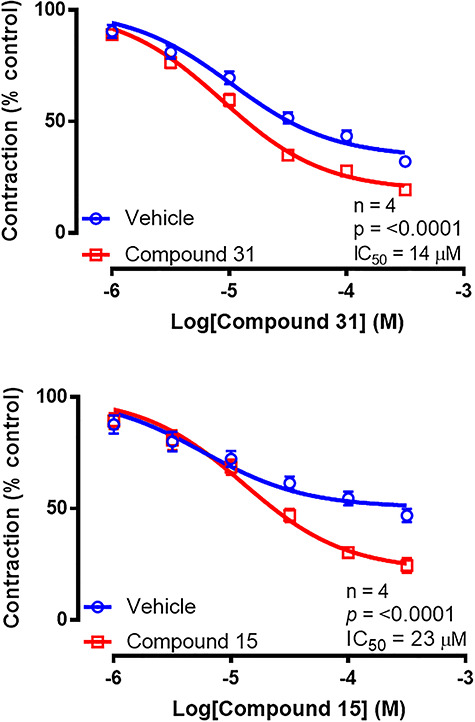
Mean inhibitory log concentration–response curves for compound 31 (1–300 μM, top), compound 15 (1–300 μM, bottom), and their corresponding vehicles on contractile responses to electrical field stimulation (60 V, 0.5 ms, 0.2 Hz) of isolated preparations of rat vas deferens. Each data point represents the mean percentage of contractions (compared to control) of tissues taken from four rats. Error bars represent SEM. Two-way repeated measures ANOVA was used to determine *P* (probability of an interaction between the treatment and concentration being due to chance) where *P*$\le$0.05 (^*^), *P*$\le$0.005 (^*^^*^), *P*$\le$0.0005 (^*^^*^^*^), and *P*$\le$0.0001 (^*^^*^^*^^*^).

Optimization efforts next focused on examining if substituents had additive effects in improving compound potency. However, with the exception of hydroxy in the ortho position with fluoro in the para position (**31**, IC_50_ = 14 (12–16) μM, see Figure 1 for structure), which provided a 3-fold improvement on either of its individual substituents (Figure 2; Table 2), all other combinations led to reduced activity (Table 2).

### Effect of washes on inhibitory effects of compounds on EFS-induced contractions

In all cases, washing of isolated rat vas deferens preparations following maximum inhibition of EFS-induced contractions by the highest concentration of test compounds, resulted in partial recovery of inhibition back to at least 80% of the control contraction height.

### Effect of prazosin on EFS-induced contractions

The addition of prazosin (300 nM) to organ baths containing compound 17 (3-dimethylbenzamido) (300 μM) reduced the residual mean EFS-induced vas deferens contractile response by 45% from 31 to 17% of the initial mean control contractions (*P* = 0.0188, *n* = 4). The addition of prazosin (300 nM) to organ baths containing the corresponding vehicle control (final bath concentration = 1.20% DMSO) reduced the residual mean EFS-induced vas deferens contractile responses by 36% from 61 to 39% of the initial mean control contractions (*P* = 0.0017, *n* = 4).

Similarly, the addition of prazosin (300 nM) to organ baths containing compound 26 (4-hydroxy) (300 μM) reduced the residual mean EFS-induced vas deferens contractile response by 33% from 24 to 16% of the initial mean control contractions (*P* = 0.0265, *n* = 4). The addition of prazosin (300 nM) to organ baths containing the corresponding vehicle control (final bath concentration = 3.36% DMSO) reduced the residual mean EFS-induced vas deferens contractile responses by 43% from 42 to 24% of the initial mean control contractions (*P* = 0.0025, *n* = 4).

### Effect of test compounds on exogenously administered αß-methylene ATP-induced contractions

Test compounds that had an IC_50_ of <100 μM underwent further testing against exogenously administered αß-methylene ATP, so that the nature and selectivity of P2X1-purinoceptor antagonism of each compound could be investigated. Exogenous administration of αß-methylene ATP (10 nM–30 μM) consistently produced concentration-dependent contractions of isolated rat vas deferens preparations.

**Figure 3:**
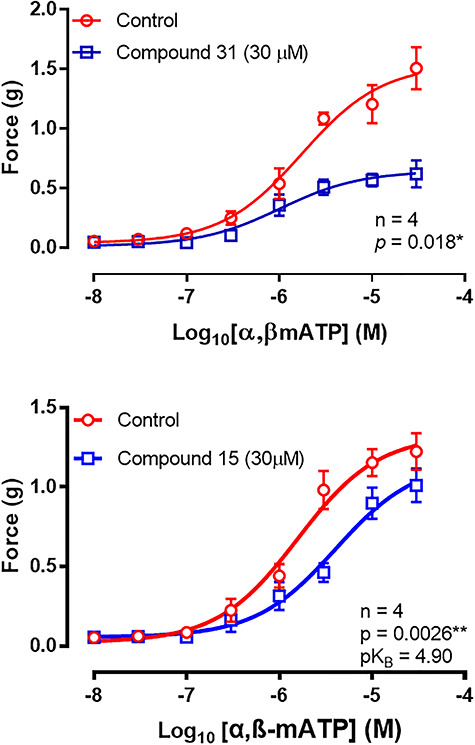
Mean log discrete concentration–response curves to αβ-methylene ATP (10 nM–30 μM) before and after administration of compound 31 (30 μM, top) or compound 15 (30 μM, bottom) on unstimulated isolated preparations of rat vas deferens. Each data point represents the mean contractile response of tissues taken from four rats. Error bars represent SEM. Two-way repeated measures ANOVA was used to determine *P* (probability of an interaction between the treatment and concentration being due to chance) where *P*$\le$0.05 (^*^), *P*$\le$0.005 (^*^^*^), *P*$\le$0.0005 (^*^^*^^*^), and *P*$\le$0.0001 (^*^^*^^*^^*^).

Compound 15 (30 μM) caused a parallel rightward shift of the mean concentration–response curve to αß-methylene ATP, with a 17% decrease in the mean peak contractile response produced by 30 μM αß-methylene ATP (Figure 3) (*P* = 0.0026, *n* = 4). A pK_B_ value of 4.9 (4.8–5.0) was calculated for compound 15. A similar parallel rightward shift (*P* = 0.0036, *n* = 4) without the decrease in maximum response was also seen with compound 26. A pK_B_ value of 4.8 (4.6–5.0) was calculated for compound 26.

Compound 31 (30 μM) caused a nonparallel rightward shift of the mean concentration–response curve to αß-methylene ATP, with a 59% decrease in the mean contractile contractile response produced by 30 μM αß-methylene ATP (Figure 3) (*P* = < 0.0001, *n* = 4). Compound 11 caused a similar nonparallel shift with a 36% depression in the mean maximal contractile response to αß-methylene ATP (*P* = <0.0001, *n* = 4).

### Effect of test compounds on contractions induced by exogenously administered noradrenaline

Several compounds were also tested with exogenously administered noradrenaline to investigate whether their antagonistic activity was selective for P2X1-purinoceptors over α_1A_-adrenoceptors or another nonspecific mechanism.

**Figure 4:**
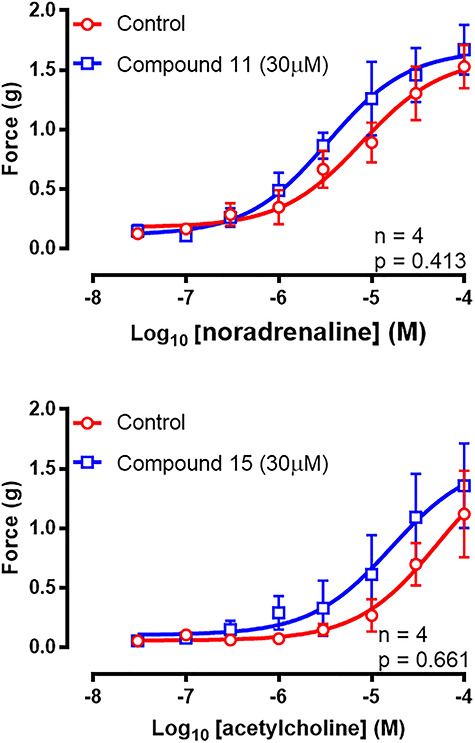
Mean log discrete concentration–response curves to noradrenaline (30 nM–100 μM) before and after administration of compound 11 (30 μM, top) or acetylcholine (10 nM–30 μM) before and after administration of compound 15 (30 μM, bottom) on unstimulated isolated preparations of rat vas deferens. Each data point represents the mean contractile response of tissues taken from four rats. Error bars represent SEM. Two-way repeated measures ANOVA was used to determine *P* (probability of an interaction between the treatment and concentration being due to chance) where *P*$\le$0.05 (^*^), *P*$\le$0.005 (^*^^*^), *P*$\le$0.0005 (^*^^*^^*^), and *P*$\le$0.0001 (^*^^*^^*^^*^).

Exogenous administration of noradrenaline (30 nM–100 μM) produced concentration-dependent contractions of the isolated rat vas deferens. None of the test compounds inhibited responses to exogenously administered noradrenaline, indicating a lack of antagonist activity at α_1A_-adrenoceptors. However, compound 11 (30 μM) produced a slight leftward shift of the mean concentration–response curve to noradrenaline while having no effect on the mean maximal contractile response produced by 100 μM noradrenaline (Figure 4) (*P* = 0.431, *n* = 4).

### Effect of test compounds on contractions induced by exogenously administered acetylcholine

Several compounds were tested against exogenously administered acetylcholine to investigate whether their antagonistic activity was selective for P2X1-purinoceptors over muscarinic cholinergic receptors or another nonspecific mechanism.

Exogenous administration of acetylcholine (30 nM–100 μM) produced concentration-dependent contractions of the isolated rat vas deferens. Compound 15 (30 μM), and all other compounds tested, had no effect on the concentration–response curves to acetylcholine (Figure 4) (*P* = 0.661, *n* = 4).

## Discussion

The generation of new contraceptive options could improve the lives of many and help control global overpopulation. Dual pharmacological antagonism of P2X1-purinoceptors and α_1A_-adrenoceptors could result in a viable male contraceptive, via the inhibition of smooth muscle contractions of the vas deferens leading to suppressed sperm transport. Given the availability of adequate α_1A_-adrenoceptor antagonists, the focus of this study was on the development of a suitable P2X1-purinoceptor antagonist.

Due to the lack of potent and selective options currently available, 33 novel compounds were synthesized and tested for P2X1-purinoceptor antagonist activity. Using compound 1 as a starting point, a series of analogues were generated with varying substituents in the ortho, meta, and para positions of the phenyl moiety.

Compound 1 was previously tested in our laboratory using the same EFS assay. It decreased the magnitude of mean EFS-induced vas deferens contractions by 18% more than its corresponding vehicle control and had an IC_50_ of 133 μM (Table 2).

Introducing different substituents to the ortho position of the phenyl moiety had little beneficial effect on the inhibitory properties of test compounds, with the exception of a hydroxyl group (11), which led to an almost 3-fold increase in activity. This suggests only a small polar substituent is suitable in this position. Conversely, the addition of substituents to the meta position had substantial effects on the inhibitory activity of test compounds. In particular, the addition of polar substituents yielded compounds with significantly improved potencies compared to compound 1. Both small electron-donating groups such as amino and hydroxy as well as electron-withdrawing groups containing a carbonyl moiety such as acetyl and methyl esters as well as methyl amides led to increased inhibitory effects on mean EFS-induced vas deferens contractions. This implies that hydrogen-bonding appears to be involved in the interaction between compounds and the receptor binding site. However, the size of the group is also crucial. Increasing the bulkiness from a dimethyl amide to morpholine amide led to a loss of inhibitory activity, suggesting that the binding pocket within the receptor binding site is small.

The addition of substituents to the para position of the phenyl moiety had varying effects on the inhibitory activity of test compounds. The addition of polar, acidic substituents resulted in compounds with high potency, while the addition of a polar, nonacidic acetamido group diminished inhibitory effects. This suggests acidity in the para position may be important for antagonist activity, potentially due to ionic bonding occurring within the receptor binding site with basic amino acids. The addition of fluoro and isobutyl groups to the para position also results in inhibitory activity (Table 2), suggesting that other nonionic interactions may be occurring at the receptor binding site. At this stage the structure–activity relationship (SAR) of para substitutions is unclear, and further investigation and optimization is required to improve the antagonist activity of test compounds.

Three di-substituted compounds displayed inhibitory activity. Compound **31** (2-hydroxy, 4-fluoro) had the greatest potency of all test compounds with an IC_50_ of 14 μM. Thus the combination of 2-hydroxy and 4-fluoro singular substitutions appeared to have an additive effect on inhibitory activity, as their independent IC_50_ values (54 and 44 μM, respectively) were not as great as that of the di-substituted compound. However, this was the only case where the activity of a di-substituted compound was markedly better than the corresponding singular substitutions. Further di-substitutions should be investigated to try and elucidate whether the result seen with compound **31** was a one-off or if other combinations can indeed improve potency in an additive manner.

The addition of prazosin (300 nM) to organ baths, in combination with our test compounds (300 μM), was able to further reduce the magnitude of mean EFS-induced contractions, resulting in a total inhibition of contractions by up to 84%. This degree of inhibition was not achieved with the addition of prazosin (300 nM) to the corresponding vehicle controls, indicating that both our test compounds and prazosin were important for the additive inhibition of contractions observed. We expect that with optimization of concentrations, dual antagonism could result in 100% inhibition of contractions. This was a clear demonstration that our novel test compounds were inhibiting the non-adrenergic/purinergic component of the EFS-induced contractions of the rat vas deferens.

Of the compounds that were selected for testing against exogenously administered αß-methylene ATP, compounds 11 and 31 displayed clear features of noncompetitive antagonism, characterized by a nonparallel rightward shift and decrease in maximal contractile response of the mean concentration–response curves to αß-methylene ATP.

In contrast, compounds 15 and 26 caused a parallel rightward shift to the mean concentration–response curve to αß-methylene ATP. Compound 26 had no effect on the mean maximal contractile response and produced a parallel rightward shift to the mean concentration–response curve to αß-methylene ATP; thus it appears to display competitive antagonism, but repeating the assay with higher concentrations of the test compound would give a better insight into its antagonistic properties. The mean concentration–response curve to αß-methylene ATP in the presence of compound 15 did not reach the same mean maximal response as the control, but this may have occurred if higher concentrations of αß-methylene ATP were able to be used. Hence it was concluded that compound 15 is also likely to be a competitive antagonist.

This observation may be important given that some of the MIPS compounds exhibited competitive antagonism, while the majority showed noncompetitive antagonism. It is entirely possible that they are binding to different sites on the P2X1-purinoceptor or in different orientations, and this may be useful for future structural studies of the P2X1-purinoceptor, should they be pursued.

The MIPS test compounds tested in this series displayed good receptor selectivity, as no compounds had any inhibitory effects on the mean concentration–response curves to either noradrenaline or acetylcholine. This suggests that the inhibitory effects of our test compounds were a result of selective antagonism at P2X1-purinoceptors, rather than some other nonselective action. Nevertheless, compound 11 produced a slight leftward shift of the mean concentration–response curve to noradrenaline, indicating a possible nonspecific off-target effect perhaps at an amine transporter.

Compound 32 was the only test compound tested against exogenously administered αß-methylene ATP which failed to shift the mean concentration–response curve. This compound required a large amount of DMSO to remain in solution, and the corresponding vehicle control caused a nonparallel rightward shift and a decrease of mean maximal contractile response of the mean concentration–response curve to αß-methylene ATP. In this case it appears that the inhibitory effects on EFS-induced contractions of the vas deferens may have been due to the DMSO present, rather than a result of compound 32. While the minimum amount of DMSO possible was used during compound dilution, poor solubility of some test compounds resulted in the use of very high amounts of DMSO being necessary in some cases. This highlights a caveat of our research, whereby the true inhibitory activity of some compounds may be masked by DMSO as a result of potential tissue degradation [29]. This appears more prevalent in the inhibitory cumulative concentration–response curve assay where tissues are constantly exposed to the solvent. Using the exogenously administered agonist concentration–response curve assay, we can better assess the effects of our compounds with less interference from DMSO, as tissues are thoroughly washed following every agonist addition. However, these assays are more time-consuming and costly as αß-methylene ATP is expensive. Further compound optimization is needed to improve solubility, particularly as an antagonist suitable for oral use in vivo should ideally be water-soluble to aid drug dissolution and absorption [30].

Rat vasa deferentia organ bath assays were selected for our research as they allow us to test our compounds in a relevant tissue sample with integrated smooth muscle, neuronal, and epithelial cells, providing a more physiologically relevant setting than a cell-based assay could provide. Inhibitory effects of P2X1-purinoceptor antagonists in recombinant cell-based assays often produce conflicting results to those obtained in isolated tissues due to the lack of ectoATPases present in recombinant systems. Using a tissue based approach from the beginning will avoid this confounding factor that would cause major complications in vivo. However, there are limitations associated with the use of rat tissue. It is possible that the results we see in rat tissue may not translate to human tissue. This is because differences between the rat and human P2X1-purinoceptor may affect drug binding, and therefore test compounds may have favorable activity depending on the species in which they are tested. There may also be differences in receptor expression, desensitization, and internalization, complicating the translation of novel drug candidates from animal to human studies and highlighting the need for therapeutically relevant tissue to be used in earlier stages of drug development. However, despite its limitations, the use of rat tissue does have several advantages. Notably, it is more consistent than human vasa deferentia samples would be. Human tissue can be incredibly variable given that samples would likely come from patients of different ages, ethnic backgrounds, and genetics. Furthermore, patients may be taking different medications and their diet, environmental factors, and lifestyle choices could all impact the results of experiments. Using tissue samples from laboratory animals, as we have done, negates all of these potential areas for variation and allows for consistency within our experiments.

Ideally, we would like to test our compounds in vivo, because although more physiologically relevant than cell-based assays, organ bath studies still exclude the complex signalling profile mediated by P2X1-purinoceptors in other parts of the body. It is therefore important to consider the potential unwanted effects which our antagonists may have in vivo. Unfortunately, we are yet to synthesize an antagonist potent enough to warrant whole animal studies. However, despite the relatively weak potency, the demonstrated activity appears pathway-selective, so the findings of this study have contributed to the generation of a structure–activity relationship (SAR) profile and will aid the development of new 2-phenyl-5,6,7,8-tetrahydroquinoxaline analogues which will hopefully have improved potency.

## Supplementary Material

Supplementary_Information_ioaa117
