# Supplementary material for: Synthesis of 2-phenyl-5,6,7,8-tetrahydroquinoxaline derivatives and screening for P2X1-purinoceptor antagonist activity in isolated preparations of rat vas deferens, for translation into a male contraceptive
Source: Biol Reprod. 2020 Jul 10;103(2):323–32. doi: 10.1093/biolre/ioaa117 (PMC7526726; doi:10.1093/biolre/ioaa117)
Supplement: Supplementary_Information_ioaa117 [file supplementary_information_ioaa117.docx]

**Instrumentation:**

*NMR spectra* were recorded on an Avance III Nanobay 400 MHz Bruker spectrometer coupled to the BACS 60 automatic sample changer. Data acquisition and processing was managed using MestReNova version 6.0.2-5475. Coupling constants (J) are recorded in Hz and the significant multiplets described by singlet (s), doublet (d), triplet (t), quartet (q), quintet (quin), sextet (sext), septet (sept), doublet of doublets (dd), doublet of doublet of doublets (ddd), triplet of doublets (td), triplet of triplets (tt), multiplet (m).

*Liquid chromatography – mass spectrometry* (LCMS) was performed using an Agilent 6100 Series Single Quad LC/MS, incorporating a photodiode array detector (214/254 nm) coupled directly to an electrospray ionisation source.

*Analytical high-performance liquid chromatography* (HPLC) was carried out on the Waters 2690 Separation Module coupled with a Waters 996 Photodiode Array Detector with a Phenomenex Luna C-8 column (100 Å, 5 μm, 150 x 4.6 mm).

*Column chromatography* was conducted using Chromatographic Silica as the stationary phase supplied by Merck.

**General Procedure A:** **Synthesis of 2-Aryl-5,6,7,8-tetrahydroquinoxaline via Palladium catalyzed Suzuki Coupling**

Glycinamide (30 mmol, 1.0 equiv.) was dissolved in methanol (60 ml) and cooled to -30 °C. Aqueous sodium hydroxide (12.5M, 1.0 equiv.) was then added. The reaction was allowed to stir for 10 minutes before a methanol solution of cyclohexadione (1M, 1.0 equiv.) was added. The reaction was stirred for 30 minutes before it was allowed to warm to -10 °C and stirred overnight. The reaction mixture was then filtered through celite to remove the solids and the volatiles were removed under reduced pressure. The residue was purified via silica gel column chromatography (eluent: CH_2_Cl_2_:methanol = 4:1).**^1^**

To a dried flask, 5,6,7,8-tetrahydroquinoxalin-2(1*H*)-one (17.8 mmol, 1.0 equiv.) and PhNTf_2_ (8.3 g, 1.3 equiv.) and DMAP (217.5 mg, 0.1 equiv.) was added to dry dichloromethane (45 ml). Triethylamine (7.5 ml, 3.0 equiv.) was then added to the reaction mixture. The reaction was allowed to stir at room temperature for 4 hours. The volatiles were removed under reduced pressure. The residue was purified via silica gel column chromatography (eluent: petroleum benzine:EtOAc = 9:1).

To a dried flask was added 5,6,7,8-tetrahydroquinoxalin-2-yl trifluoromethanesulfonate (0.5 mmol, 1.0 equiv.), aryl boronic acid (1.3 equiv.), cesium carbonate (407.3 mg, 2.5 equiv.) and Pd(PPh_3_)_4_ (28.9 mg, 0.05 equiv.). A degassed mixture of dioxane/water (4:1, 5 ml) was then added. This was then heated to 80 ⁰C and stirred for 16 hours at which point reaction completion was determined by TLC/LCMS analysis. The reaction mixture was then filtered through Celite to remove the catalyst and the volatiles were removed under reduced pressure. The residue was purified via silica gel column chromatography (eluent: petroleum benzine:EtOAc = 3:1 for 3-5, 7-10, 13-19, 22-24, 27, 28, 33, 34; CH_2_Cl_2_:methanol = 9:1 for 6, 11, 12, 25, 26, 29-32). .

**5,6,7,8-Tetrahydroquinoxalin-2-yl Trifluoromethanesulfonate (iii)**

Yellow oil (3.7 g, 74%). **^1^H NMR** (400 MHz, chloroform-*d*) δ 8.30 (s, 1H), 3.12 – 2.72 (m, 4H), 1.95 (m, 4H). **^13^C NMR** (101 MHz, chloroform-*d*) δ 154.0, 152.3, 150.5, 133.5, 31.5, 31.4, 22.3, 22.0.

**2-(2-Fluorophenyl)-5,6,7,8-tetrahydroquinoxaline (3)**

Yellow oil (101.9 mg, 89%). **^1^H NMR** (400 MHz, chloroform-*d*) δ 8.79 (d, *J* = 2.8 Hz, 1H), 7.94 (td, *J* = 7.8, 1.9 Hz, 1H), 7.45 – 7.33 (m, 1H), 7.28 – 7.25 (m, 1H), 7.17 (ddd, *J* = 11.2, 8.2, 1.2 Hz, 1H), 3.07 – 2.95 (m, 4H), 1.97 (ddd, *J* = 6.8, 4.0, 2.8 Hz, 4H). **^13^C NMR** (101 MHz, chloroform-*d*) δ 161.7, 159.2, 153.2, 151.1, 146.3, 141.4, 141.3, 131.0, 130.9, 130.8, 130.8, 124.8, 124.7, 124.6, 116.4, 116.2, 32.1, 31.4, 22.6, 22.5. **Analytical HPLC** tr = 6.033, >99% (254nm). **LCMS** (ESI) *m/z* [M+H]^+^ 229.2, tr = 6.67.

**2-(2-Chlorophenyl)-5,6,7,8-tetrahydroquinoxaline (4)**

Yellow solid (49.0 mg, 40%). mp: 76 – 78 °C. **^1^H NMR** (400 MHz, chloroform-*d*) δ 8.66 (s, 1H), 7.62 – 7.54 (m, 1H), 7.51 – 7.46 (m, 1H), 7.43 – 7.33 (m, 2H), 3.03 (t, *J* = 2.7 Hz, 4H), 1.98 (dq, *J* = 6.8, 3.0 Hz, 4H). **^13^C NMR** (101 MHz, chloroform-*d*) δ 153.0, 151.0, 149.6, 141.5, 136.2, 132.4, 131.6, 130.2, 127.3, 32.0, 31.5, 22.6, 22.5. **Analytical HPLC** tr = 6.075, >99% (254nm). **LCMS** (ESI) *m/z* [M+H]^+^ 245.2, tr = 6.68.

**2-(2-Methoxyphenyl)-5,6,7,8-tetrahydroquinoxaline (5)**

Yellow oil (91.6 mg, 76%). **^1^H NMR** (400 MHz, chloroform-*d*) δ 8.82 (s, 1H), 7.75 (dd, *J* = 7.6, 1.8 Hz, 1H), 7.39 (ddd, *J* = 8.2, 7.3, 1.7 Hz, 1H), 7.08 (td, *J* = 7.5, 1.0 Hz, 1H), 7.00 (dd, *J* = 8.3, 1.0 Hz, 1H), 3.06 – 2.96 (m, 4H), 1.98 – 1.93 (m, 4H). **^13^C NMR** (101 MHz, chloroform-*d*) δ 157.1, 152.4, 150.0, 148.7, 142.6, 131.0, 130.4, 126.3, 121.2, 111.3, 55.6, 32.1, 31.5, 22.7, 22.7. **Analytical HPLC** tr = 5.528, >99% (254nm). **LCMS** (ESI) *m/z* [M+H]^+^ 241.0, tr = 3.468.

**2-(5,6,7,8-Tetrahydroquinoxalin-2-yl)phenol (6)**

Yellow solid (34.1 mg, 30%). mp: 131 – 133 °C. **^1^H NMR** (400 MHz, chloroform-*d*) δ 8.98 (s, 1H), 7.85 (dd, *J* = 8.0, 1.6 Hz, 1H), 7.34 (ddd, *J* = 8.6, 7.2, 1.6 Hz, 1H), 7.03 (dd, *J* = 8.3, 1.2 Hz, 1H), 6.95 (ddd, *J* = 8.2, 7.2, 1.3 Hz, 1H), 3.07 – 2.98 (m, 4H), 1.98 (h, *J* = 3.2 Hz, 4H).

**^13^C NMR** (101 MHz, chloroform-*d*) δ 159.6, 150.1, 149.9, 149.3, 137.4, 132.1, 125.8, 119.4, 118.6, 117.0, 31.4, 31.1, 22.4, 22.2. **Analytical HPLC** tr = 6.184, >99% (254nm). **LCMS** (ESI) *m/z* [M+H]^+^ 227.2, tr = 6.67.

**2-(5,6,7,8-Tetrahydroquinoxalin-2-yl)benzonitrile (7)**

Yellow solid (20.1 mg, 17%). mp: 93 – 95 °C. **^1^H NMR** (400 MHz, chloroform-*d*) δ 8.72 (s, 1H), 7.89 – 7.76 (m, 2H), 7.71 (td, *J* = 7.7, 1.3 Hz, 1H), 7.54 (td, *J* = 7.4, 1.1 Hz, 1H), 3.15 – 3.03 (m, 4H), 1.99 (p, *J* = 3.6 Hz, 4H). **^13^C NMR** (101 MHz, chloroform-*d*) δ 153.0, 153.0, 147.8, 140.6, 140.3, 134.4, 132.9, 129.6, 129.1, 118.3, 111.4, 31.8, 22.5. **Analytical HPLC** tr = 5.347, >99% (254nm). **LCMS** (ESI) *m/z* [M+H]^+^ 236.2, tr = 6.15.

**1-(2-(5,6,7,8-Tetrahydroquinoxalin-2-yl)phenyl)ethan-1-one (8)**

Yellow oil (91.1 mg, 72%). **^1^H NMR** (400 MHz, chloroform-*d*) δ 8.57 (d, *J* = 0.9 Hz, 1H), 7.62 (ddd, *J* = 7.5, 1.5, 0.6 Hz, 1H), 7.58 – 7.55 (m, 1H), 7.53 (dd, *J* = 7.4, 1.6 Hz, 1H), 7.49 (dd, *J* = 7.4, 1.4 Hz, 1H), 3.06 – 2.93 (m, 5H), 1.95 (p, *J* = 3.4 Hz, 4H). **^13^C NMR** (101 MHz, chloroform-*d*) δ 203.3, 152.1, 151.4, 150.0, 141.3, 140.0, 135.9, 130.6, 129.3, 129.0, 127.8, 31.6, 30.4, 23.9, 22.6, 22.5. **Analytical HPLC** tr = 5.006, >99% (254nm). **LCMS** (ESI) *m/z* [M+H]^+^ 253.1, tr = 4.57.

**2-(m-Tolyl)-5,6,7,8-tetrahydroquinoxaline (9)**

Yellow solid (102.6 mg, 91%). mp: 44 – 46 °C. **^1^H NMR** (400 MHz, chloroform-*d*) δ 8.72 (s, 1H), 7.79 (s, 1H), 7.74 (d, *J* = 7.9 Hz, 1H), 7.37 (t, *J* = 7.6 Hz, 1H), 7.28 – 7.21 (m, 1H), 3.01 (dt, *J* = 15.9, 5.1 Hz, 5H), 2.44 (s, 3H), 1.96 (t, *J* = 3.5 Hz, 4H). **^13^C NMR** (101 MHz, chloroform-*d*) δ 152.3, 151.1, 149.9, 138.9, 138.6, 136.9, 130.0, 128.8, 127.5, 123.9, 32.2, 31.7, 22.7, 21.6. **Analytical HPLC** tr = 5.707, >99% (254nm). **LCMS** (ESI) *m/z* [M+H]^+^ 225.1, tr = 3.38.

**2-(3-Chlorophenyl)-5,6,7,8-tetrahydroquinoxaline (10)**

Yellow solid (42.9 mg, 35%). mp: 99 – 101 °C. **^1^H NMR** (400 MHz, chloroform-*d*) δ 8.72 (s, 1H), 8.00 (dt, *J* = 2.5, 1.1 Hz, 1H), 7.84 (ddd, *J* = 5.8, 3.3, 1.7 Hz, 1H), 7.43 – 7.39 (m, 2H), 3.06 – 3.01 (m, 4H), 2.03 – 1.94 (m, 4H). **^13^C NMR** (101 MHz, chloroform-*d*) δ 153.7, 150.9, 148.9, 138.1, 136.9, 135.2, 130.3, 129.7, 127.0, 124.7, 32.1, 31.1, 22.5, 22.4. **Analytical HPLC** tr = 6.876, >99% (254nm). **LCMS** (ESI) *m/z* [M+H]^+^ 245.2, tr = 7.09.

**3-(5,6,7,8-Tetrahydroquinoxalin-2-yl)phenol (11)**

Yellow solid (62.4 mg, 56%). mp: 188 – 190 °C. **^1^H NMR** (400 MHz, DMSO-*d*_6_) δ 9.59 (s, 1H), 8.85 (s, 1H), 7.59 – 7.45 (m, 2H), 7.30 (ddd, *J* = 8.2, 7.3, 0.9 Hz, 1H), 6.85 (ddd, *J* = 8.0, 2.3, 1.2 Hz, 1H), 2.99 – 2.87 (m, 5H), 1.89 (p, *J* = 3.4 Hz, 4H). **^13^C NMR** (101 MHz, DMSO-*d*_6_) δ 158.3, 152.0, 151.5, 148.8, 138.9, 138.0, 130.4, 117.6, 116.9, 113.6, 32.0, 31.6, 22.6. **Analytical HPLC** tr = 4.821, >99% (254nm). **LCMS** (ESI) *m/z* [M+H]^+^ 227.2, tr = 5.77.

**3-(5,6,7,8-Tetrahydroquinoxalin-2-yl)aniline (12)**

Yellow solid (17.0 mg, 15%). mp: 207 – 209 °C. **^1^H NMR** (400 MHz, DMSO-*d*_6_) δ 8.91 (s, 1H), 7.80 – 7.65 (m, 2H), 7.43 (td, *J* = 7.8, 1.5 Hz, 1H), 7.12 (d, *J* = 7.9 Hz, 1H), 2.93 (dt, *J* = 11.6, 5.2 Hz, 4H), 1.90 (p, *J* = 3.4 Hz, 4H). **^13^C** NMR (101 MHz, DMSO-*d*_6_) δ 152.3, 152.0, 148.1, 138.9, 137.8, 130.6, 121.9, 121.1, 117.9, 32.0, 31.6, 22.5, 22.5. **Analytical HPLC** tr = 3.715, >99% (254nm). **LCMS** (ESI) *m/z* [M+H]^+^ 226.1, tr = 2.49.

**3-(5,6,7,8-Tetrahydroquinoxalin-2-yl)benzonitrile (13)**

Yellow solid (70.9 mg, 60%). mp: 116 – 117 °C. **^1^H NMR** (400 MHz, chloroform-*d*) δ 8.75 (s, 1H), 8.33 (t, *J* = 1.8 Hz, 1H), 8.21 (ddd, *J* = 7.9, 1.8, 1.2 Hz, 1H), 7.71 (dt, *J* = 7.7, 1.4 Hz, 1H), 7.60 (td, *J* = 7.8, 0.6 Hz, 1H), 3.03 (dd, *J* = 6.1, 2.9 Hz, 4H), 2.05 – 1.95 (m, 4H). **^13^C NMR** (101 MHz, chloroform-*d*) δ 153.2, 152.5, 147.3, 138.1, 138.1, 132.5, 130.7, 130.5, 129.7, 118.5, 113.3, 32.2, 31.7, 22.6, 22.5. **Analytical HPLC** tr = 6.021, >99% (254nm). **LCMS** (ESI) *m/z* [M+H]^+^ 236.1, tr = 3.47.

**1-(3-(5,6,7,8-Tetrahydroquinoxalin-2-yl)phenyl)ethan-1-one (14)**

Yellow solid (102.8 mg, 81%). mp: 86 – 88 °C. **^1^H NMR** (400 MHz, chloroform-*d*) δ 8.79 (d, *J* = 0.9 Hz, 1H), 8.56 (td, *J* = 1.8, 0.6 Hz, 1H), 8.18 (ddd, *J* = 7.8, 1.8, 1.1 Hz, 1H), 8.02 (ddd, *J* = 7.8, 1.8, 1.1 Hz, 1H), 7.59 (td, *J* = 7.8, 0.6 Hz, 1H), 3.07 – 2.97 (m, 4H), 2.68 (s, 3H), 2.00 – 1.94 (m, 4H). **^13^C NMR** (101 MHz, chloroform-*d*) δ 197.9, 152.7, 151.9, 148.6, 138.8, 137.8, 137.5, 131.2, 129.2, 129.0, 126.6, 32.2, 31.8, 26.8, 22.7. **Analytical HPLC** tr = 5.202, >99% (254nm). **LCMS** (ESI) *m/z* [M+H]^+^ 253.1, tr = 2.99.

**Methyl 3-(5,6,7,8-Tetrahydroquinoxalin-2-yl)benzoate (15)**

Yellow solid (114.3 mg, 85%). mp: 75 – 77 °C. **^1^H NMR** (400 MHz, chloroform-*d*) δ 8.82 – 8.74 (m, 1H), 8.63 (d, *J* = 1.8 Hz, 1H), 8.26 – 8.17 (m, 1H), 8.10 (dq, *J* = 7.8, 1.4 Hz, 1H), 7.56 (td, *J* = 7.8, 2.3 Hz, 1H), 4.04 – 3.91 (m, 3H), 3.09 – 2.89 (m, 5H), 1.99 – 1.92 (m, 4H). **^13^C NMR** (101 MHz, chloroform-*d*) δ 166.8, 152.7, 151.7, 148.7, 138.5, 137.3, 131.1, 130.9, 130.3, 129.1, 127.8, 52.2, 32.2, 31.7, 22.6. **Analytical HPLC** tr = 6.068, >99% (254nm). **LCMS** (ESI) *m/z* [M+H]^+^ 269.1, tr = 3.18.

***N*-Methyl-3-(5,6,7,8-tetrahydroquinoxalin-2-yl)benzamide (16)**

Yellow solid (69.7 mg, 43%). mp: 152 – 154 °C. **^1^H NMR** (400 MHz, chloroform-*d*) δ 8.79 (s, 1H), 8.38 (t, *J* = 1.8 Hz, 1H), 8.10 (dt, *J* = 7.8, 1.4 Hz, 1H), 7.86 – 7.82 (m, 1H), 7.56 (t, *J* = 7.8 Hz, 1H), 3.06 (d, *J* = 4.9 Hz, 3H), 3.04 – 2.98 (m, 4H), 1.97 (p, *J* = 3.7 Hz, 4H). **^13^C NMR** (101 MHz, chloroform-*d*) δ 167.9, 151.9, 148.6, 138.8, 137.3, 135.5, 129.4, 129.2, 127.7, 125.0, 32.2, 31.7, 26.9, 22.7. **Analytical HPLC** tr = 4.850, >99% (254nm). **LCMS** (ESI) *m/z* [M+H]^+^ 324.1, tr = 2.02.

***N*,*N*-Dimethyl-3-(5,6,7,8-tetrahydroquinoxalin-2-yl)benzamide (17)**

Yellow solid (83.2 mg, 59%). mp: 118 – 120 °C. **^1^H NMR** (400 MHz, chloroform-*d*) δ 8.75 (s, 1H), 8.30 – 7.92 (m, 2H), 7.72 – 7.41 (m, 2H), 3.15 (s, 4H), 3.03 (d, *J* = 6.3 Hz, 6H), 1.97 (h, *J* = 3.1 Hz, 4H). **^13^C NMR** (101 MHz, chloroform-*d*) δ 171.2, 152.5, 151.7, 148.7, 138.7, 137.2, 137.0, 128.9, 127.7, 127.7, 125.5, 39.6, 35.4, 32.2, 31.7, 22.6. **Analytical HPLC** tr = 5.134, >99% (254nm). **LCMS** (ESI) *m/z* [M+H]^+^ 282.0, tr = 4.37.

**Morpholino(3-(5,6,7,8-tetrahydroquinoxalin-2-yl)phenyl)methanone (18)**

Yellow solid (79.4 mg, 49%). mp: 94 – 96 °C. **^1^H NMR** (400 MHz, chloroform-*d*) δ 8.75 (s, 1H), 8.30 – 7.95 (m, 2H), 7.64 – 7.40 (m, 2H), 3.73 (d, *J* = 52.4 Hz, 8H), 3.02 (d, *J* = 7.3 Hz, 4H), 1.97 (ddd, *J* = 6.8, 4.4, 2.8 Hz, 4H). **^13^C NMR** (101 MHz, chloroform-*d*) δ 170.1, 152.6, 151.9, 148.5, 138.7, 137.5, 136.0, 129.1, 128.1, 127.7, 125.6, 66.9, 32.2, 31.7, 22.7. **Analytical HPLC** tr = 5.059, >99% (254nm). **LCMS** (ESI) *m/z* [M+H]^+^ 324.2, tr = 2.66.

**2-(4-Fluorophenyl)-5,6,7,8-tetrahydroquinoxaline (22)**

Yellow solid (106.9 mg, 93%). mp: 89 – 91 °C. **^1^H NMR** (400 MHz, chloroform-*d*) δ 8.70 (s, 1H), 8.03 – 7.91 (m, 3H), 7.17 (t, *J* = 8.7 Hz, 2H), 3.08 – 2.95 (m, 4H), 2.00 – 1.93 (m, 4H). **^13^C NMR** (101 MHz, chloroform-*d*) δ 165.0, 162.5, 152.6, 151.0, 148.9, 138.1, 128.7, 128.6, 116.1, 115.8, 32.2, 31.5, 22.7, 22.6. **Analytical HPLC** tr = 6.168, >99% (254nm). **LCMS** (ESI) *m/z* [M+H]^+^ 229.2, tr = 6.02.

**2-(4-Chlorophenyl)-5,6,7,8-tetrahydroquinoxaline (23)**

Yellow solid (41.6 mg, 34%). mp: 116 – 118 °C. **^1^H NMR** (400 MHz, chloroform-*d*) δ 8.71 (s, 1H), 7.93 (d, *J* = 8.7 Hz, 2H), 7.45 (d, *J* = 8.6 Hz, 2H), 3.05 – 2.98 (m, 4H), 2.00 – 1.91 (m, 5H). **^13^C NMR** (101 MHz, chloroform-*d*) δ 153.5, 150.5, 149.1, 136.9, 135.9, 134.8, 129.2, 128.0, 32.1, 31.1, 22.5, 22.4. **Analytical HPLC** tr = 6.791, >99% (254nm). **LCMS** (ESI) *m/z* [M+H]^+^ 245.0, tr = 3.93.

**2-(4-(Trifluoromethyl)phenyl)-5,6,7,8-tetrahydroquinoxaline (24)**

Yellow oil (83.5 mg, 59%). **^1^H NMR** (400 MHz, chloroform-*d*) δ 8.77 (s, 1H), 8.25 – 8.03 (m, 2H), 7.83 – 7.67 (m, 2H), 3.04 (d, *J* = 6.2 Hz, 4H), 1.98 (p, *J* = 3.5 Hz, 4H). **^13^C NMR** (101 MHz, chloroform-*d*) δ 153, 152.3, 148.2, 138.7, 127.0, 125.8, 125.8, 32.2, 31.7, 23.9, 22.6, 22.6. **Analytical HPLC** tr = 6.528, >99% (254nm). **LCMS** (ESI) *m/z* [M+H]^+^ 283.1, tr = 5.50.

**4-(5,6,7,8-Tetrahydroquinoxalin-2-yl)phenol (26)**

Yellow solid (27.2 mg, 24%). mp: 230 – 232 °C. **^1^H NMR** (400 MHz, DMSO-*d*_6_) δ 9.91 (s, 1H), 8.80 (s, 1H), 7.91 (d, *J* = 8.7 Hz, 2H), 6.87 (d, *J* = 8.7 Hz, 2H), 2.87 (d, *J* = 14.5 Hz, 5H), 1.86 (q, *J* = 3.3 Hz, 4H). **^13^C NMR** (101 MHz, DMSO-*d*_6_) δ 159.2, 151.8, 150.1, 149.0, 138.1, 128.3, 127.5, 116.2, 32.0, 31.4, 22.7. **Analytical HPLC** tr = 4.724, >99% (254nm). **LCMS** (ESI) *m/z* [M+H]^+^ 227.3, tr = 5.74.

**4-(5,6,7,8-Tetrahydroquinoxalin-2-yl)benzonitrile (27)**

Yellow solid (100.4 mg, 85%). mp: 121 – 123 °C. **^1^H NMR** (400 MHz, chloroform-*d*) δ 8.78 (s, 1H), 8.17 – 8.08 (m, 2H), 7.86 – 7.70 (m, 2H), 3.10 – 2.97 (m, 4H), 1.98 (dq, *J* = 6.8, 3.7, 3.2 Hz, 4H). **^13^C NMR** (101 MHz, chloroform-*d*) δ 153.4, 152.6, 147.6, 140.9, 138.3, 132.7, 127.2, 118.6, 112.9, 32.2, 31.6, 22.5, 22.5, 14.2. **Analytical HPLC** tr = 6.902, >99% (254nm). **LCMS** (ESI) *m/z* [M+H]^+^ 236.3, tr = 6.43.

**1-(4-(5,6,7,8-Tetrahydroquinoxalin-2-yl)phenyl)ethan-1-one (28)**

Yellow solid (58.3 mg, 46%). mp: 121 – 123 °C. **^1^H NMR** (400 MHz, chloroform-*d*) δ 8.79 (s, 1H), 8.11 – 8.04 (m, 4H), 3.09 – 2.98 (m, 4H), 2.65 (s, 3H), 2.02 – 1.93 (m, 4H). **^13^C NMR** (101 MHz, chloroform-*d*) δ 197.6, 152.8, 141.2, 139.0, 137.4, 128.9, 126.8, 32.2, 31.8, 26.7, 22.7. **Analytical HPLC** tr = 5.838, >99% (254nm). **LCMS** (ESI) *m/z* [M+H]^+^ 253.0, tr = 3.41.

**4-(5,6,7,8-Tetrahydroquinoxalin-2-yl)benzoic acid (29)**

Yellow solid (14.2 mg, 11%). mp: 231 – 233 °C. **^1^H NMR** (400 MHz, DMSO-*d*_6_) δ 9.02 (s, 1H), 8.21 (d, *J* = 8.1 Hz, 2H), 8.06 (d, *J* = 8.1 Hz, 2H), 2.95 (dt, *J* = 16.2, 5.0 Hz, 5H), 1.90 (p, *J* = 3.2 Hz, 4H). **^13^C NMR** (101 MHz, DMSO-*d*_6_) δ 196.3, 152.5, 152.5, 147.7, 140.6, 139.5, 130.3, 126.9, 32.0, 31.7, 22.6. **Analytical HPLC** tr = 5.213, >99% (254nm). **LCMS** (ESI) *m/z* [M+H]^+^ 255.2, tr = 5.77.

**4-(5,6,7,8-Tetrahydroquinoxalin-2-yl)benzene-1,3-diol (30)**

Yellow solid (29.1 mg, 24%). mp: 240 – 242 °C. **^1^H NMR** (400 MHz, DMSO-*d*_6_) δ 9.01 (s, 1H), 7.87 (d, *J* = 8.7 Hz, 1H), 6.39 (dd, *J* = 8.7, 2.4 Hz, 1H), 6.31 (d, *J* = 2.4 Hz, 1H), 3.00 – 2.81 (m, 3H), 1.86 (p, *J* = 3.6 Hz, 4H). **^13^C NMR (**101 MHz, DMSO-*d*_6_) δ 160.9, 160.6, 149.6, 149.3, 148.5, 139.0, 128.8, 110.2, 108.2, 103.9, 31.3, 31.2, 22.5, 22.3. **Analytical HPLC** tr = 5.136, >99% (254nm). **LCMS** (ESI) *m/z* [M+H]^+^ 242.3, tr = 4.89.

**5-Fluoro-2-(5,6,7,8-tetrahydroquinoxalin-2-yl)phenol (31)**

Yellow solid (55.0 mg, 45%). mp: 179 – 181 °C. **^1^H NMR** (400 MHz, chloroform-*d*) δ 8.90 (s, 1H), 8.21 – 7.49 (m, 1H), 6.85 – 6.34 (m, 2H), 2.98 (d, *J* = 6.5 Hz, 5H), 1.97 (t, *J* = 3.5 Hz, 4H). **^13^C NMR** (101 MHz, chloroform-*d*) δ 166.0, 163.5, 161.7, 161.6, 150.6, 149.0, 148.5, 138.0, 127.2, 127.1, 113.7, 107.0, 106.8, 105.4, 105.2, 31.4, 31.3, 22.5, 22.3. **Analytical HPLC** tr = 6.448, >99% (254nm). **LCMS** (ESI) *m/z* [M+H]^+^ 245.1, tr = 3.32.

**2-Isobutyl-5-(5,6,7,8-tetrahydroquinoxalin-2-yl)phenol (32)**

Yellow solid (99.2 mg, 71%). mp: 174 – 176 °C. **^1^H NMR** (400 MHz, chloroform-*d*) δ 8.71 (d, *J* = 0.8 Hz, 1H), 7.51 – 7.38 (m, 2H), 7.21 – 7.13 (m, 1H), 3.01 (t, *J* = 5.8 Hz, 4H), 2.54 (d, *J* = 7.2 Hz, 2H), 1.96 (tq, *J* = 4.6, 2.9, 2.3 Hz, 5H), 0.93 (dd, *J* = 11.6, 6.6 Hz, 6H). **^13^C NMR** (101 MHz, chloroform-*d*) δ 154.5, 152.9, 150.3, 149.9, 135.3, 131.9, 129.8, 118.7, 113.5, 39.2, 32.0, 31.1, 28.8, 22.5, 22.5, 22.5. **Analytical HPLC** tr = 6.959, >99% (254nm). **LCMS** (ESI) *m/z* [M+H]^+^ 279.0, tr = 5.80.

**1-(2-Fluoro-5-(5,6,7,8-tetrahydroquinoxalin-2-yl)phenyl)ethan-1-one (33)**

Yellow solid (67.8 mg, 50%). mp: 73 – 75 °C. **^1^H NMR** (400 MHz, chloroform-*d*) δ 8.75 (s, 1H), 8.47 (dd, *J* = 7.0, 2.4 Hz, 1H), 8.19 (ddd, *J* = 8.6, 4.7, 2.5 Hz, 1H), 7.29 – 7.22 (m, 1H), 3.06 – 2.96 (m, 4H), 2.70 (d, *J* = 4.9 Hz, 3H), 1.97 (td, *J* = 4.1, 2.1 Hz, 4H). **^13^C NMR** (101 MHz, chloroform-*d*) δ 195.5, 195.5, 164.2, 161.6, 153.1, 151.3, 148.0, 137.6, 133.3, 133.0, 132.9, 129.0, 128.9, 126.2, 126.0, 117.6, 117.4, 32.1, 31.4, 22.6, 22.5. **Analytical HPLC** tr = 5.907, >99% (254nm). **LCMS** (ESI) *m/z* [M+H]^+^ 271.1, tr = 5.14.

**Methyl 2-Fluoro-5-(5,6,7,8-tetrahydroquinoxalin-2-yl)benzoate (34)**

Yellow solid (47.4 mg, 33%). mp: 65 – 67 °C. **^1^H NMR** (400 MHz, chloroform-*d*) δ 8.75 (s, 1H), 8.55 (dd, *J* = 6.9, 2.5 Hz, 1H), 8.18 (ddd, *J* = 8.7, 4.6, 2.5 Hz, 1H), 7.26 (s, 1H), 3.98 (s, 3H), 3.03 (d, *J* = 4.9 Hz, 4H), 1.97 (p, *J* = 3.7 Hz, 4H). **^13^C NMR** (101 MHz, chloroform-*d*) δ 164.6, 164.6, 163.8, 161.2, 152.6, 151.8, 147.6, 138.3, 133.1, 133.0, 132.7, 132.6, 130.4, 130.4, 119.1, 119.0, 117.8, 117.6, 52.5, 32.1, 31.7, 22.6. **Analytical HPLC** tr = 6.062, >99% (254nm). **LCMS** (ESI) *m/z* [M+H]^+^ 287.0, tr = 5.20.

**General Procedure B: Synthesis of 2-Aryl-5,6,7,8-tetrahydroquinoxaline via Palladium catalyzed direct arylation^2^**

In a dried flask, K_2_CO_3_ (276.4 mg, 2.0 equiv.) and the aryl bromide (1 mmol, 1.0 equiv.) were added to 5,6,7,8-tetrahydroquinoxaline *N-*oxide (450.5 mg, 3.0 equiv.) in dry dioxane (10 ml). The flask was then put under vacuum and purged under nitrogen for 5 minutes. The reaction mixture was then degassed for 10 minutes. Pd(OAc)_2_ (11.2 mg, 0.05 equiv.) and HP(*t*-Bu)_3_BF_4_ (43.5 mg, 0.15 equiv.) were added to the reaction mixture. This was then heated to 110⁰C and stirred for 24 hours at which point reaction completion was determined by TLC/LCMS analysis. The reaction mixture was then filtered through Celite to remove the catalyst and the volatiles were removed under reduced pressure. The residue was purified via silica gel column chromatography (eluent: CH_2_Cl_2_:methanol = 9:1).

Ammonium formate (630 mg, 20.0 equiv.) was added to the *N-*oxide (0.5 mmol, 1.0 equiv.) in MeOH (5 ml), Palladium on carbon (53.2 mg, 0.1 equiv.) was added to the solution and the reaction mixture was left to stir at room temperature for 24 hours. Reaction completion was determined by TLC analysis and the solution was filtered through Celite to remove the catalyst and the volatiles were removed under reduced pressure. The residue was purified via silica gel column chromatography (eluent: petroleum benzine:EtOAc = 4:1).

**2-(o-Tolyl)-5,6,7,8-tetrahydroquinoxaline 1-Oxide (v-2)**

Red oil (120.4 mg, 50%). **^1^H NMR** (400 MHz, chloroform-*d*) δ 8.29 (s, 1H), 7.39 (td, *J* = 7.5, 1.6 Hz, 1H), 7.34 – 7.27 (m, 2H, H), 7.23 (dd, *J* = 7.5, 1.4 Hz, 1H), 3.02 – 2.92 (m, *J* = 12.3, 5.8 Hz, 4H), 2.21 (s, 3H), 1.99 – 1.88 (m, 4H). **^13^C NMR** (101 MHz, chloroform-*d*) δ 155.7, 144.5, 143.6, 143.4, 138.7, 130.3, 130.2, 130.0, 129.9, 126.0, 32.0, 24.1, 22.0, 21.7, 19.7. **LCMS** (ESI) *m/z* [M+H]^+^ 241.0, tr = 3.15.

**2-(4-Isopropylphenyl)-5,6,7,8-tetrahydroquinoxaline 1-Oxide (v-20)**

Brown solid (150.0 mg, 42%). mp: 165 – 167 °C. **^1^H NMR** (400 MHz, chloroform-*d*) δ 8.41 (s, 1H), 7.70 (dt, *J* = 8.4, 1.8 Hz, 2H), 7.35 (dt, *J* = 8.2, 1.6 Hz, 2H), 3.03 – 2.88 (m, 5H), 1.99 – 1.85 (m, 4H), 1.28 (d, *J* = 6.9 Hz, 6H). **^13^C NMR** (101 MHz, chloroform-*d*) δ 154.9, 151.0, 143.9, 143.7, 142.1, 129.4, 127.5, 126.7, 34.3, 32.0, 24.3, 24.0, 21.9, 21.8. **LCMS** (ESI) *m/z* [M+H]^+^ 269.2, tr = 3.45.

**2-(4-Isobutylphenyl)-5,6,7,8-tetrahydroquinoxaline 1-Oxide (v-21)**

White solid (365.8 mg, 49%). mp: 135 – 137 °C. **^1^H NMR** (400 MHz, chloroform-*d*) δ 8.42 (s, 1H), 7.70 (d, *J* = 8.3 Hz, 2H), 7.27 (d, *J* = 8.0 Hz, 2H), 3.00 – 2.93 (m, 4H), 2.53 (d, *J* = 7.2 Hz, 2H), 1.98 – 1.86 (m, 5H), 0.93 (d, *J* = 6.6 Hz, 6H). **^13^C NMR** (101 MHz, chloroform-*d*) δ 154.9, 143.9, 143.6, 142.0, 129.3, 129.1, 127.4, 45.5, 32.0, 30.3, 24.2, 22.5, 22.0, 21.8. **LCMS** (ESI) *m/z* [M+H]^+^ 283.0, tr = 3.56.

**2-(4-Isopropoxyphenyl)-5,6,7,8-tetrahydroquinoxaline 1-Oxide (v-25)**

White solid (96.5 mg, 27%). mp: 141 – 143 °C. **^1^H NMR** (400 MHz, chloroform-*d*) δ 8.41 (s, 1H), 7.75 (dt, *J* = 9.0, 2.1 Hz, 2H), 6.98 (dt, *J* = 8.9, 2.1 Hz, 2H), 4.63 (sept, *J* = 6.1 Hz, 1H), 3.01 – 2.92 (m, 4H), 1.99 – 1.85 (m, 4H), 1.37 (d, *J* = 6.1 Hz, 6H). **^13^C NMR** (101 MHz, chloroform-*d*) δ 159.4, 154.5, 143.7, 143.6, 141.8, 130.9, 121.9, 115.7, 70.2, 31.9, 24.3, 22.2, 22.0, 21.8. **LCMS** (ESI) *m/z* [M+H]^+^ 285.2, tr = 3.34.

**2-(o-Tolyl)-5,6,7,8-tetrahydroquinoxaline (2)**

Yellow oil (28.3 mg, 48%). **^1^H NMR** (400 MHz, chloroform-*d*) δ 8.43 (s, 1H), 7.41 – 7.37 (m, 1H), 7.35 – 7.27 (m, 3H), 3.05 – 2.97 (m, 4H), 2.38 (s, 3H), 2.01 – 1.94 (m, 4H). **^13^C NMR** (101 MHz, chloroform-*d*) δ 152.4, 152.2, 150.8, 141.7, 137.4, 136.3, 131.1, 129.9, 128.9, 126.2, 32.2, 31.9, 22.8, 20.4. **Analytical HPLC** tr = 6.667, >99% (254nm). **LCMS** (ESI) *m/z* [M+H]^+^ 225.0, tr = 3.45.

**2-(4-Isopropylphenyl)-5,6,7,8-tetrahydroquinoxaline (20)**

Yellow solid (53.2 mg, 51%). mp: 103 – 105 °C. **^1^H NMR** (400 MHz, chloroform-*d*) δ 8.71 (s, 1H), 7.89 (dt, *J* = 8.3, 1.9 Hz, 2H), 7.34 (dt, *J* = 8.1, 1.6 Hz, 2H), 3.05 – 2.91 (m, 5H), 2.00 – 1.91 (m, 4H), 1.28 (d, *J* = 6.9 Hz, 6H). **^13^C NMR** (101 MHz, chloroform-*d*) δ 152.4, 150.8, 150.4, 150.0, 138.8, 134.7, 127.1, 126.9, 34.1, 32.3, 31.8, 24.0, 22.9. **Analytical HPLC** tr = 8.308, >99% (254nm). **LCMS** (ESI) *m/z* [M+H]^+^ 253.0, tr = 3.79.

**2-(4-Isobutylphenyl)-5,6,7,8-tetrahydroquinoxaline (21)**

Yellow solid (112.6 mg, 70%). mp: 98 – 100 °C. **^1^H NMR** (400 MHz, chloroform-*d*) δ 8.71 (s, 1H), 7.87 (dt, *J* = 8.3, 1.8 Hz, 2H), 7.26 (d, *J* = 8.2 Hz, 2H), 3.05 – 2.95 (m, 4H), 2.53 (d, *J* = 7.2 Hz, 2H), 1.99 – 1.93 (m, 4H), 1.89 (sept, *J* = 6.7 Hz, 1H), 0.92 (d, *J* = 6.6 Hz, 6H). **^13^C NMR** (101 MHz, chloroform-*d*) δ 152.4, 150.9, 150.0, 143.3, 138.9, 134.6, 129.9, 126.7, 45.4, 32.4, 31.8, 30.4, 22.9, 22.5. **Analytical HPLC** tr = 8.911, >99% (254nm). **LCMS** (ESI) *m/z* [M+H]^+^ 267.0, tr = 3.94.

**2-(4-Isopropoxyphenyl)-5,6,7,8-tetrahydroquinoxaline (25)**

White solid (61.3 mg, 84%). mp: 96 – 98 °C. **^1^H NMR** (400 MHz, chloroform-*d*) δ 8.68 (s, 1H), 7.91 (dt, *J* = 8.9, 2.1 Hz, 2H), 6.99 (dt, *J* = 8.9, 2.1 Hz, 2H), 4.63 (sept, *J* = 6.0 Hz, 1H), 3.06 – 2.97 (m, 4H), 1.99 – 1.92 (m, 4H), 1.37 (d, *J* = 6.1 Hz, 6H). **^13^C NMR** (101 MHz, chloroform-*d*) δ 159.2, 152.2, 150.3, 149.7, 138.3, 129.4, 128.2, 116.3, 70.1, 32.3, 31.7, 22.9, 22.1. **Analytical HPLC** tr = 7.617, >99% (254nm). **LCMS** (ESI) *m/z* [M+H]^+^ 269.0, tr = 3.65.

**References**

1. Brill, E., & Schultz, H. P. (1963). Quinoxaline Studies. XII. Stereodirective Synthesis of cis- and trans-Decahydroquinoxalines and cis- and trans-decahydroquinoxalones-2*. *Journal of Organic Chemistry, 29*, 579-581.

2. Leclerc, J, & Fagnou, K. (2006). Palladium-Catalyzed Cross-Coupling Reactions of Diazine *N*-Oxides with Aryl Chlorides, Bromides, and Iodides*. *Angewandte Chemie International Edition, 45*(46), 7781-7786.
